# **Bile** c**ell‑**f**ree DNA:** a g**ame** c**hanger for** m**anagement of** b**iliary** t**ract** c**ancer?**

**DOI:** 10.1016/j.esmoop.2022.100389

**Published:** 2022-02-02

**Authors:** A. Rizzo, A.D. Ricci, G. Brandi

**Affiliations:** 1 Struttura Semplice Dipartimentale di Oncologia Medica per la Presa in Carico Globale del Paziente Oncologico "Don Tonino Bello", I.R.C.C.S. Istituto Tumori "Giovanni Paolo II", Bari, Italy; 2 Department of Experimental, Diagnostic and Specialty Medicine, S. Orsola-Malpighi University Hospital, Bologna, Italy

Recent years have witnessed the advent of molecular profiling in biliary tract cancer (BTC), and new techniques and technologies have led to the identification of a variety of molecular alterations.^1^ Preliminary studies have suggested a high correspondence between mutational profile in BTC tissue and bile, and the detection of tumor-specific genetic aberrations with utilization of bile cell-free DNA (cfDNA) is under evaluation, although this strategy has not yet been implemented into clinical routine.^2^ In *ESMO Open*, Gou and colleagues report the results of a prospective study carried out in a Chinese institution to assess the role of bile as a liquid biopsy medium in patients with BTC^3^; interestingly, the authors highlighted that bile cfDNA was superior to plasma cfDNA in the detection of tumor-related alterations.

However, we have some concerns that might affect the interpretation of this trial.

First, few data are available regarding patients included in the study. For example, it is not specified whether some patients with BTC had a history of primary sclerosing cholangitis, which is the major cause of cholangiocarcinoma in several geographical areas; similarly, fluke infection was not reported. Based on these premises, we believe the cohort representativeness may be compromised. Second, the trial included a widely varied patient population from a single institute and the total number of BTCs analyzed was relatively small. In our view, the small number of patients precluded from reporting a reliable association between distinct copy number variations and different BTC subtypes.

Third, plasma-based liquid biopsy has reported a high rate of type I error (false positive) due to the age-related phenomenon of clonal hematopoiesis, causing the accumulation of nonmalignant mutations and hesitating in the presence of genetically distinct subpopulations of white blood cells. Because the majority of plasma-based cfDNA derives from peripheral blood cells and clonal hematopoiesis is highly prevalent in both healthy individuals and patients with cancer, this potential ‘mutational background noise’ should be considered when interpreting liquid biopsy results.^4^^,^^5^ We invite the authors to share their opinion on these remarks.

Despite these limitations, we believe the authors are to be commended for this interesting trial on a timely topic in BTC management. In the near future, multicenter, large-scale, well-designed clinical trials are needed to validate bile-based cfDNA analysis.
